# Glioblastoma Primary Cells Retain the Most Copy Number Alterations That Predict Poor Survival in Glioma Patients

**DOI:** 10.3389/fonc.2021.621432

**Published:** 2021-04-26

**Authors:** Chia-Hua Chen, Ya-Jui Lin, You-Yu Lin, Chang-Hung Lin, Li-Ying Feng, Ian Yi-Feng Chang, Kuo-Chen Wei, Chiung-Yin Huang

**Affiliations:** ^1^ Molecular Medicine Research Center, Chang Gung University, Taoyuan, Taiwan; ^2^ Department of Neurosurgery, Chang Gung Memorial Hospital, Linkou Medical Center, Taoyuan, Taiwan; ^3^ The Graduate Institute of Biomedical Sciences, Chang Gung University, Taoyuan, Taiwan; ^4^ Graduate Institute of Clinical Medicine, National Taiwan University College of Medicine, Taipei, Taiwan; ^5^ Department of Ophthalmology, Chang Gung Memorial Hospital, Linkou Medical Center, Taoyuan, Taiwan; ^6^ School of Medicine, Chang Gung University, Taoyuan, Taiwan; ^7^ Department of Neurosurgery, New Taipei Municipal TuCheng Hospital, New Taipei City, Taiwan

**Keywords:** primary cell, glioma, glioblastoma, prognostic factors, copy number alterations

## Abstract

Gliomas are solid tumors that originate from glial cells in the brain or spine and account for 74.6% of malignant primary central nervous system tumors worldwide. As patient-derived primary cells are important tools for drug screening and new therapy development in glioma, we aim to understand the genomic similarity of the primary cells to their parental tumors by comparing their whole-genome copy number variations and expression profile of glioma clinicopathologic factors. We found that the primary cells from grade II/III gliomas lost most of the gene copy number alterations (CNAs), which were mainly located on chromosome 1p and 19q in their parental tumors. The glioblastoma (GBM) primary cells preserved 83.7% of the gene CNAs in the parental GBM tumors, including chromosome 7 gain and 10q loss. The CNA gains of *LINC00226* and *ADAM6* and the chromosome 16p11 loss were reconstituted in primary cells from both grade II/III gliomas and GBMs. Interestingly, we found these CNAs were correlated to overall survival (OS) in glioma patients using the Merged Cohort LGG and GBM dataset from cBioPortal. The gene CNAs preserved in glioma primary cells often predicted poor survival, whereas the gene CNAs lost in grade II/III primary cells were mainly associated to better prognosis in glioma patients. Glioma prognostic factors that predict better survival, such as *IDH* mutations and 1p/19q codeletion in grade II/III gliomas, were lost in their primary cells, whereas methylated *MGMT* promoters as well as *TERT* promoter mutations were preserved in GBM primary cells while lost in grade II/III primary cells. Our results suggest that GBM primary cells tend to preserve CNAs in their parental tumors, and these CNAs are correlated to poor OS and predict worse prognosis in glioma patients.

## Introduction

Gliomas are brain tumors that result from abnormal growth of glial cells in the central nervous system and are characterized by their complex origin that give rise to multiple tumor subgroups and various histology. The WHO 2016 classification of gliomas emphasized the importance of tumor histology, *IDH* mutations and 1p/19q codeletion for categorizing gliomas (1). The WHO grade II and grade III gliomas are mainly astrocytomas (As) and oligodendrogliomas (ODGs) derived from astrocytes and oligodendrocytes, respectively, and ODG is characterized by 1p/19q codeletion. GBMs are highly malignant WHO grade IV gliomas that usually recur within one year after resection, and the five-year relative survival post diagnosis is 6.8% (2). Current glioma therapies lack effectiveness despite intensive medical care due to the heterogeneous origin of gliomas and blockage of the blood-brain barrier. Recent studies have worked towards the development of new therapeutic targets and drug delivery methods as well as the establishment of personalized precision medicine.

Several molecular markers have been known to associate with patient survival and tumor grouping in gliomas. The *IDH* mutations are strongly associated with patient survival as patients with mutations have a higher survival rate (3). Patients with WHO grade II/III gliomas are often positive for the *IDH* mutation (>80%), whereas most GBM patients are *IDH* wild-type (4). The 1p/19q codeletion, a classification marker of ODGs, is positively associated with patient response to chemotherapy as well as patient survival, in which WHO grade II/III patients with the codeletion have better prognoses (5–7). *MGMT* promoter methylation is also associated with resistance to chemotherapy with alkylating agents as the methylated promoter predicts better prognosis and longer survival (8, 9). *TERT* promoter mutation is commonly present in ODGs (78%) and *IDH*-wildtype GBMs (83%) and is considered as a potential grouping marker in glioma (5, 10). Although many molecules have been found to correlate with patient survival and treatment outcome, the WHO 2016 glioma classification was still mainly based on tumor histology, and the molecular mechanisms regulating the formation and progression of gliomas remain largely unknown.

Patient-derived xenograft (PDX) in mouse and primary cells from fresh tumor specimens are often used to evaluate new treatment efficacy during therapeutic development and clinical trials, and also plays an important role for drug screening and pathogenesis-molecular mechanism investigating (11). Therefore, the similarity of the mutational landscape and expression profile between original tumors and their derived primary cells is crucial to the patient-derived primary cell as a screening model that represents a population of tumors from different patients. As the tumor microenvironment is complex and unique in each tumor, multiple culture conditions have been used to preserve the maximum genetic identity between tumors and primary cell population (12). Although studies have confirmed that tumor-derived stem cells share more similarities to original tumors in genotype and RNA profile than traditional tumor cell lines grown in fetal bovine serum containing medium, tumor stem cell lines have low establishment success rate and high maintenance costs (12, 13). In contrast, even though traditional cultured glioma cell lines exhibit less similarity to patient tumors, they are easier to obtain, cost less, and are adherent cells with the ability to proliferate rapidly, which makes them suitable for cell modification and functional tests (13). The patient-derived cell lines cultured in traditional growth medium provide a compromise among similarity to original tumor, cost of cell culture, and facilitation of treatment, modification, and functional test of cells.

Previous studies have mainly focused on patient-derived stem cells or PDXs from GBMs, whereas little is known about the characteristics of WHO grade II/III primary cells and its similarities between tumors and their primary cells cultured in traditional growth medium. Here, we aim to understand the preservation status of tumor DNA aberrances in primary cells from tumors of different WHO grades and histology by comparing the whole-genome copy number changes between tumors and its patient-derived cells. We freshly collected primary cells from patient specimens, cultured in fetal bovine serum-containing growth medium, and compare the retention of copy number alterations (CNAs) from tumors to primary cells. Our findings indicated that primary cells from GBMs preserved most of the gene CNAs in tumors that are involved in cellular growth, movement, development, and interaction. Furthermore, the presence of these gene CNAs in tumors predicted poor survival in glioma patients.

## Materials and Methods

### Collection of Patient Specimens

Glioma patients who received brain tumor surgery at the Linkou Chang Gung Memorial Hospital, Taiwan, between February 2014 and July 2015 were enrolled in this study. Patient sample collection and usage were approved by the Chang Gung Medical Foundation Institutional Review Board (102-4005B, 102-4023B, and 99-0812B); written consents were obtained from patients prior to sample collection. Tumor tissue were stored in liquid nitrogen or preserved in culture medium for primary cell isolation immediately after resection. Primary cells from 14 patients were successfully cultured from removed tumor tissue, and the origins of the cultured primary cells to their corresponding tumors were confirmed by short tandem repeat (STR) tests. Eleven paired tissues and cells were selected for OncoScan CNV microarray test based on tissue availability, DNA quality, and cell condition. Tumor grading and pathology was determined based on morphology, arrangement of cells in tumors, *IDH1/2* status, and 1p/19q codeletion. The *IDH1/2* mutations were determined by PCR and Sanger sequencing. The 1p/19q status was determined by OncoScan microarrays. Details of patient information are listed in the **Table 1**.

**Table 1:** The information of patients who received brain tumor resection in Linkou Chang Gung Memorial Hospital from Feb. 2014 to Jul. 2015.

Patient No.	WHO grade	Age at diagnosed	Sex	Pathology	Status
W919	II	29	female	oligodendroglioma	newly diagnosed
W933	II	58	male	diffuse astrocytoma	recurrent
W937	II	40	female	oligodendroglioma	newly diagnosed
W946	II	28	male	diffuse astrocytoma	newly diagnosed
W928	III	36	female	anaplastic oligodendroglioma	recurrent
W950	III	45	female	anaplastic oligodendroglioma	newly diagnosed
W952	III	31	male	anaplastic astrocytoma	newly diagnosed
W802	IV	62	male	glioblastoma	newly diagnosed
W909	IV	58	male	glioblastoma	recurrent
W935	IV	73	female	glioblastoma	newly diagnosed
W958	IV	84	male	glioblastoma	newly diagnosed

### Isolation and Culture of Primary Cells

Glioma primary cell separation was performed according to previous reports with some modifications (12, 14). Glioma cells were dissociated from tumors by trypsinization, then the primary cells were cultured in DMEM-F12 (12400-024, Gibco) with 10% fetal bovine serum (10438-026, Gibco), 100 IU/ml penicillin, and 100 μg/ml streptomycin (15140-122, Gibco) in a humidified tissue culture incubator at 37°C and 5% CO_2_ atmosphere. Surviving primary cells after five passages were subject to STR analysis to confirm the origin with their corresponding patient, and primary cells no later than ten passages were used for detection and analyses of DNA mutations, copy number variations, and prognostic factor status.

### Detection of IDH1/2 Mutations

Tumor DNA was extracted and subjected to PCR for amplification of DNA fragments containing IDH1 codon 132 and IDH2 codon 140 and 172. PCR products were then sequenced to determine their IDH1/2 status. Detailed PCR protocols and primer sequences were described in **Supplementary Information**.

### Detection of TERT Promoter Mutations

*TERT* promoter mutation assay was performed according to previous studies (5). Briefly, tumor DNA was subjected to PCR for amplification of *TERT* promoter fragments containing two mutation hot spots C250T and C228T. PCR products were then sequenced to determine their mutation status. Detailed protocols and primer sequences were described in **Supplementary Information**.

### Measuring the MGMT Methylation Status

To analyze the methylation of *MGMT* promoter, bisulfite conversion of denatured genomic DNA was performed using EZ DNA Methylation-Gold Kit (cat. D5006; Zymo Research), followed by PCR amplification. Methylation ratio of the *MGMT* promoter was determined according to the methylation status of five CpG sites in region +17 to +39 in exon 1 of the MGMT gene by pyrosequencing using PyroMark Q24 CpG MGMT (cat#970032; Qiagen). Average methylation percentage above 10% was considered methylated and below 10% as unmethylated.

### OncoScan CNV FFPE Assay

Genomic DNA was extracted from tumors and primary cells. Tissues or cells were lysed and homogenized in Cell lysis Solution (Qiagen, #158908) followed by addition of proteinase K, RNase A, and Protein Precipitation Solution (Qiagen, #158912) for removing protein and RNA. Genomic DNA was precipitated by isopropanol, washed with ethanol, air dried, and rehydrated in TE buffer. The concentration and quality of DNA was determined using Qubit dsDNA quantification assays (Thermo Fisher Scientific), and the DNA was prepared at 12 ng/μL for 6.6 μL/well per sample. Copy number variations were then determined using OncoScan CNV Plus Reagent Kit (Thermo Fisher Scientific, Cat. No. 902294) according to the manufacturers’ instructions. The arrays were washed, stained, and scanned with the GeneChip Scanner 3000 7G and GeneChip Fluidics Station 450. Array fluorescence intensity data (CEL files) were generated by Affymetrix® GeneChip® Command Console® (AGCC) Software version 4.0 and processed to OSCHP files and QC metrics by OncoScan Console software version 1.1.034. Copy number variation, loss of heterozygosity, and the percentage of aberrant cell were calculated using Nexus Express for OncoScan 3 with the TuScan algorithm.

### Analyses of the Relationship Between Samples

The unrooted phylogenetic tree of gene CNV total events was generated with R package “ape” with maximum parsimony method and default parameters. The Principal Component Analysis (PCA) graph was determined based on the total events of gene CNV using the built-in R function prcomp() with default setting and was visualized using R package “factoextra” to build a ggplot2-based graph.

### Data Analyses

Total gene CNV profile of tumor and paired primary cells were analyzed to identify CNA preservation and lost in primary cells. The gene CNAs that occurred in more than two tumor samples were included in the analyses. A tumor gene CNA was defined as fully or partially preserved if it was passed and preserved in its corresponding primary cells for all or at least 60% of the analyzed tumor samples, respectively. Similarly, a tumor gene CNA was defined as fully or partially lost if it was lost in its corresponding primary cells for all or at least 60% of the analyzed tumor samples, respectively. Pathway analyses were performed used QIAGEN Ingenuity Pathway Analysis (IPA) (QIAGEN, Jan 2020).

### Survival Analysis

Survival analysis was performed using the Merged Cohort of LGG and GBM (TCGA, Cell 2016) dataset and copy number alteration as well as OS information were downloaded from cBioPortal. Among the total 1122 cases in the obtained dataset including both GBMs and grade II/III gliomas (LGG, low-grade glioma in the dataset), 1017 cases consisted of complete gene CNV and overall survival information and were included in further analyses. Approximately 83.6% (2026/2424), 82.3% (660/802), 57% (4/7), and 76.5% (648/847) of the CNAs we detected for CNA loss lost, CNA loss preserved, CNA gain lost, and CNA gain preserved, respectively, were also present in the genomes of the glioma patients (**Table 6**). The R package “survival” and “survminer” were used to evaluate the correlation between CNAs and patient OS with Cox proportional hazards regression analysis, perform log-rank test and determine the hazard ratios (HRs) for the univariate Cox regression. HR represents the ratio of risk of death for patients with the CNA compared to patients without the CNA. P-value < 0.05 for the log-rank test were considered statistically significant.

## Results

### The Copy Number Variations in Glioma Specimens and Paired Primary Cells

**Figure 1:**
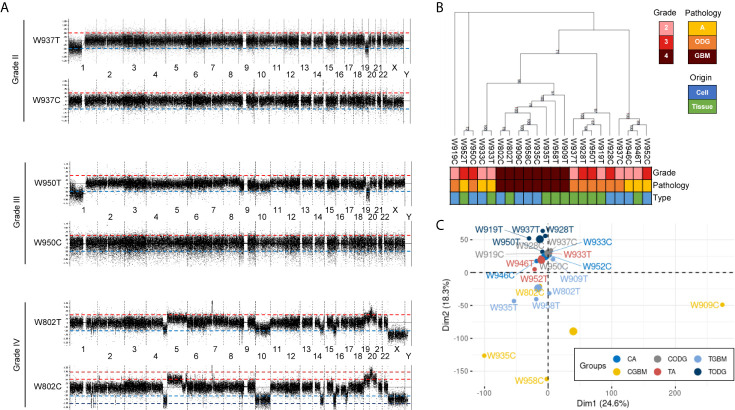
Basic information of copy number variation (CNV) in glioma patient specimens and patient-derived primary cells. **(A)** Representative whole-genome views of CNV in different grades of glioma patient specimens and paired primary cells. Chromosome numbers are labeled between paired tissue and cell. T, patient tissue. C, patient-derived primary cell. **(B)** A maximum parsimony phylogenic tree for examining the relationship among patient samples and paired primary cells based on the differences of CNA events. A, astrocytoma; ODG, oligodendroglioma; GBM, glioblastoma. **(C)** PCA graph of sample similarity analysis based on CNA differences. Larger circles are mean points of each group. CA, astrocytoma-derived primary cells; CGBM, GBM-derived primary cells; CODG, oligodendroglioma-derived primary cells; TA, astrocytoma specimens; TODG, oligodendroglioma specimens; TGBM, GBM specimens.

Paired primary cells were successfully isolated from 11 glioma patient specimens, including 4 (W919, W933, W937, W946), 3 (W928, W950, W952), and 4 (W802, W909, W935, W958) cells from WHO grade II, WHO grade III, and WHO grade IV tumors, respectively (**Table 1**). Among all primary cells, only the 4 WHO grade IV cells were able to continue dividing rapidly for more than 30 passages. Each tissue-primary cell pair all have similarities greater than 95% (**Supplementary Table 1**). The OncoScan FFPE Assay was carried out to identify the genome-wide copy number, loss of heterozygosity (LOH) and somatic mutations. A total of 16,774 genes with CNA and 36,081 CNA events were identified among all 11 tumor tissues (**Table 2**). Chromosome 1p and 19q codeletion was frequently observed in grade II and grade III tumors but not in their primary cells (**Figure 1A**). The CNAs in GBM and their primary cells were more diverse than in grade II/III samples. The CNA phylogenetic tree indicated GBM tissue and primary cells were closely clustered together, whereas ODG tumor tissues clustered together separate from their primary cell counterparts (**Figure 1B**). Furthermore, PCA analysis demonstrated that the tumors of astrocytoma as well as primary cells other than GBM were located closer to each other, whereas GBM primary cells were more dispersed (**Figure 1C**). The results indicated GBM gene CNAs were dramatically different than grade II/III samples regardless of the sample sources. Therefore, GBM and grade II/III groups were analyzed separately in further analyses.

**Table 2:** Summary of the CNA presence in patient specimens and the CNA preservation in patient-derived primary cells.

	Glioma (N=11)	Grade II/III (N=7)	GBM (N=4)
Total CNA	36081	19409	16661
Amp	10163	1695	8462
Loss	25918	17714	8199
Total gene	16774	10271	11812
Amp	7209	1334	6031
Loss	12845	9332	6136
Preserved CNA	17431 (48.3%)	4093 (21.1%)	13329 (80.0%)
Amp	6663 (65.6%)	424 (25.0%)	6235 (73.7%)
Loss	10768 (41.6%)	3669 (20.7%)	7094 (86.5%)
Preserved gene	11707 (69.8%)	3152 (30.7%)	9891 (83.7%)
Amp	5126 (71.1%)	294 (22.0%)	4840 (80.3%)
Loss	7393 (57.6%)	2858 (30.6%)	5195 (84.7%)

### Most of the Gene CNAs Were Located on Chromosome 1p and 19q in Grade II/III Tumors and Lost in Primary Cells

**Figure 2:**
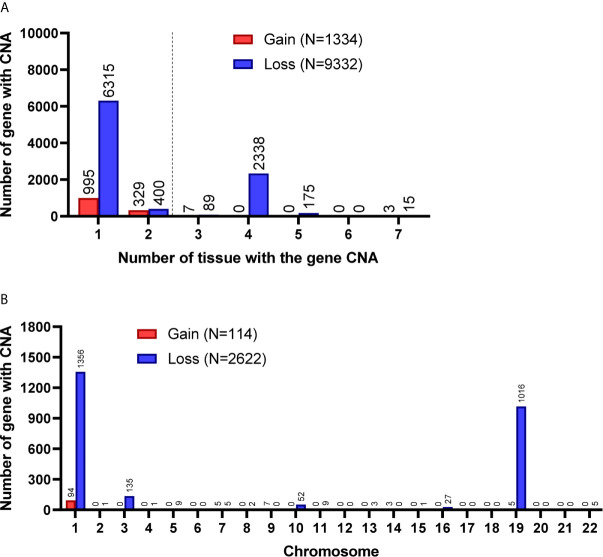
Gene CNAs in parental WHO grade II/III glioma specimens. **(A)** Number of tumors contains genes with CNA in patient specimens. Genes with CNA in more than two tissue specimens were enrolled in the following studies. **(B)** The distribution of genes with CNA on chromosomes.

**Figure 3:**
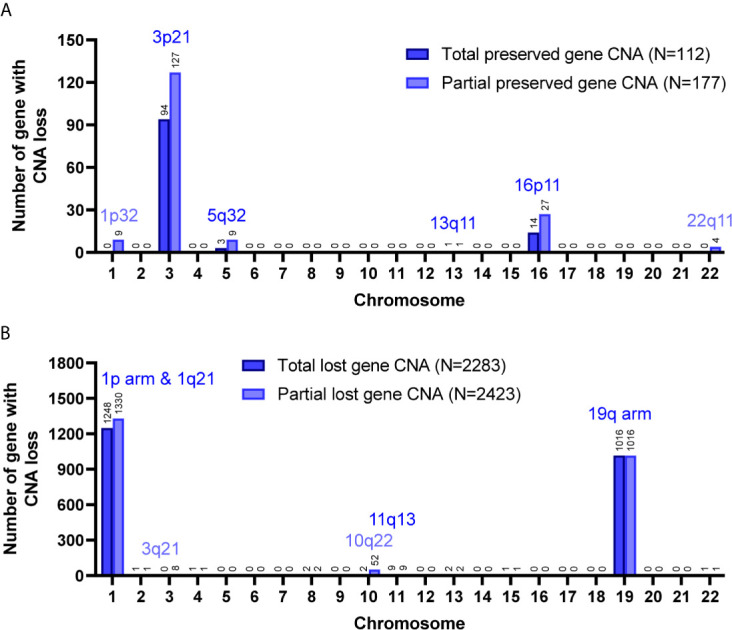
Preservation status of genes with CNA loss in cultured primary cells compared to the parental grade II/III tumors. Total, the CNAs of targeted genes in patient specimens were all preserved/lost in their primary cells. Partial, at least 60% of primary cells preserved or lost the targeted gene CNAs. The chromosome distribution of the preserved **(A)** or lost **(B)** gene CNAs in primary cells.

Among the 7 grade II/III tumors, there were 19,409 CNA events involving 10,271 genes, and the majority of CNA events were copy number loss with 17,714 events (91.3%) involving 9,332 genes (**Table 2**). However, most of the events occurred independently in 7 grade II/III tumors (**Figure 2A**). Genes with CNA presence in more than 2 tumor samples were located mainly on chromosome 1 (1,450), 3 (135), 10 (52), 16 (25), and 19 (1021) (**Figure 2B**). Results show only 3 CNA gain and 177 CNA loss were partially preserved in primary cells, indicating preservation rates of 2.6% and 6.8% for CNA gain and loss, respectively (**Table 3** and **Figure 3A**). The genes with preserved CNA gain were located on chromosome 14q32, whereas the genes with preserved CNA loss were mainly located on chromosome 3p21 and 16p11. In contrast to CNA preserved in primary cells, the partial gene CNA lost rate including gene CNA gain and loss in grade II/III samples was as high as 88.8% (2430/2736), which indicated most of the gene CNAs occurred in grade II/III tumors were lost in primary cells. Most of the gene CNA lost events were CNA losses located on chromosome 1p arm, 10q22, and 19q arm (**Figure 3B**), whereas only 7 gene CNA gain located on chromosome 9q34 were lost in primary cells (**Table 3**). A detailed list of preserved and lost gene CNAs in grade II/III gliomas was provided in **Supplementary Table 2**. The results indicated that the majority of the CNAs in glioma grade II/III tumors were CNA losses on chromosome 1p and 19q, and most were lost in tumor-derived primary cells.

**Table 3:** Preservation and lost of genes with CNA gain in patient-derived primary cells compared to their original tumors in the grade II/III group.

Amplified Gene name	Chr. location	Preservation status of CNVs
FAM30A	14q32	Total preserved
ADAM6	14q32	Partial preserved
LINC00226	14q32	Partial preserved
EHMT1	9q34	Partial lost
EHMT1-IT1	9q34	Partial lost
MIR602	9q34	Partial lost
LOC100133077	9q34	Partial lost
CACNA1B	9q34	Partial lost
LOC101928786	9q34	Partial lost
TUBBP5	9q34	Partial lost

### GBM Primary Cells Preserved the Most Gene CNAs

**Figure 4:**
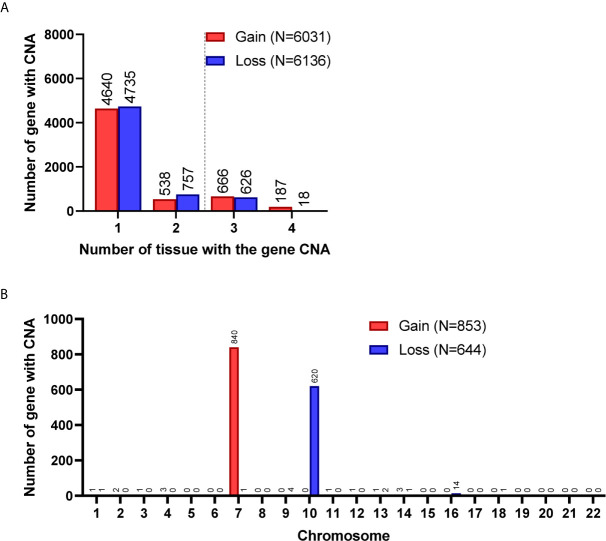
Gene CNAs in parental GBM specimens. **(A)** Number of tumors contains genes with CNA in patient specimens. Genes with CNA in more than two tissue specimens were enrolled in the following studies. **(B)** The distribution of genes with CNA on chromosomes.

**Figure 5:**
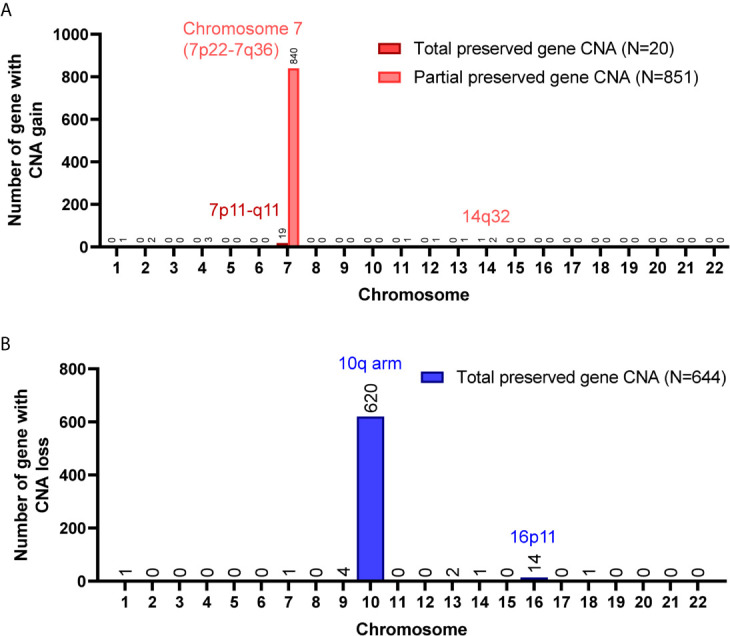
Chromosome distribution of genes with CNA preserved in GBM primary cells. **(A)** The chromosome distribution of genes with CNA gain preserved in primary cells. **(B)** The chromosome distribution of genes with CNA loss preserved in primary cells. Total, the targeted gene CNAs in patient specimens were all preserved/lost in their primary cells. Partial, at least 60% of primary cells preserved or lost the targeted gene CNAs.

A total of 8,462 CNA gain and 8,199 CNA loss were detected among the 4 GBM tissues (**Table 2**). Most of the gene CNAs were single event that only occurred in one of the 4 GBM samples (**Figure 4A**). The CNA gain events involving 853 genes were mainly located on chromosome 7, whereas 644 genes with CNA loss were mainly located on chromosome 10 (**Figure 4B**). *MIR7976*, located on chromosome 3q21, was the single gene with a total lost CNA (**Table 4**). The genes with CNA gain on chromosome 7 (**Figure 5A**) as well as the genes with CNA loss on chromosome 10q arm and 16p11 (**Figure 5B**) in tumors were all preserved in their primary cells. The detailed gene CNA list was provided in **Supplementary Table 3**. Together, a total of 9,891 genes with CNA in GBM tumors were preserved in tumor-derived primary cells, the preservation rate is 83.7% (**Table 2**), which suggests that most of the gene CNAs in GBM tissues were preserved in their primary cells.

**Table 4:** Lost of gene CNA gain in patient-derived primary cells compared to their original tumors in the GBM group.

Amplified Gene name	Chr. location	Preservation status of CNAs
MIR7976	3q21	Total lost

### Cell Growth Related Signaling Pathways Were the Important Features in Gene CNA Preservation

To understand the correlation between the disease and the preservation of gene CNAs in primary cells, pathway analysis examining molecular and biological functions of the genes was performed using IPA software (QIAGEN). The molecular and cellular functions as well as the disease and biological functions that significantly correlated to the genes with CNA preserved or lost in primary cells were determined and listed by ascending P value in **Supplementary Tables 4** and **5** (P <0.05), respectively. Among the top ten biological functions of the genes with CNA preserved or lost in primary cells, cellular development, cellular growth and proliferation, small molecule biochemistry, and cell-to-cell signaling and interaction were observed in all grade II/III CNA-preserved, grade II/III CNA-lost, and GBM CNA-preserved groups (**Table 5**). Biological functions that associated to cell growth, including cellular development, growth and proliferation, cell death and survival, and cell cycle, accounted for half of the intersected functions among all three groups. Disease and biological function analyses results suggest that cell growth, proliferation, and survival were upregulated whereas cell differentiation, apoptosis, and death were downregulated in both CNA-preserved groups, especially in GBM primary cells (**Supplementary Table 5** and **Supplementary Figure 1**). The results indicated that glioma primary cells predominately preserve gene CNAs that regulate cell growth, whereas preserved gene CNAs were suggested to contribute towards increased cell growth as well as decreased cell death.

**Table 5:** The top molecular and biological functions associated to CNA genes in grade II/III CNA-preserved, grade II/III CNA-lost, and GBM CNA-preserved group.

Category	Involved #Molecules	Grade II/IIICNA-lost	Grade II/III CNA-preserved	GBM CNA-preserved
Cellular Development	198	V	V	V
Cellular Growth and Proliferation	174	V	V	V
Small Molecule Biochemistry	164	V	V	V
Cell-To-Cell Signaling and Interaction	114	V	V	V
Cell Death and Survival	163	V		V
Cellular Assembly and Organization	83	V		V
Cellular Movement	173		V	V
Cell Cycle	68		V	V

### Gene CNAs Preserved or Lost in Primary Cells Correlated With Overall Survival in Glioma Patients

**Figure 6:**
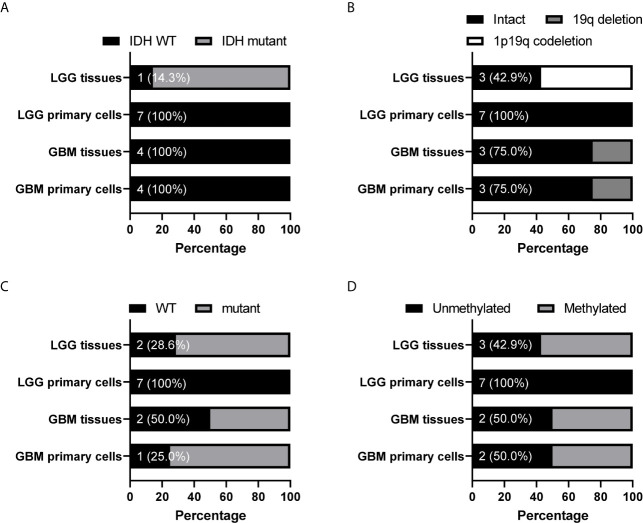
Preservation status of glioma molecular markers in patient-derived primary cells compared to their original specimens in the grade II/III and GBM groups. The status of *IDH* mutation **(A)**, chromosome 1 p arm and chromosome 19 q arm deletion **(B)**, *TERT* promoter mutation **(C)**, and *MGMT* promoter methylation **(D)** in 7 grade II/III and 4 GBM paired tumors and primary cells were determined. The numbers indicated the number of tissue or cell with wild type *IDH* or *TERT* promoter, intact 1p/19q, or unmethylated *MGMT* promoter. WT, wild type.

Because tumor cell growth contributes towards tumor growth and is correlated to cancer patient survival, we next examined the correlation between the gene CNAs and patient OS in glioma using the Merged Cohort of LGG and GBM (TCGA, Cell 2016) dataset from cBioPortal. The survival analysis result indicated majority of CNAs detected (99.5%, 81.8%, 100%, and 99.9% for CNA loss lost, CNA loss preserved, CNA gain lost, and CNA gain preserved, respectively) were correlated to glioma patient OS (log-rank test, P <0.05) (**Table 6**). A total of 2015 genes with CNA loss lost in primary cells were correlated to patient OS (log-rank test, P <0.05) and most of them with HR <1, whereas 540 genes with preserved CNAs were correlated to OS and mainly HR >1. For genes with CNA-gain preserved in primary cells, 647 out of 648 genes with the CNA information were correlated to patient OS and the HRs of the patients positive of the gene CNA gains were all greater than 1. The results showed that patients positive of gene CNAs which were lost in primary cells have HR less than one, whereas patients positive of gene CNAs which were preserved in primary cells have HR greater than one. Moreover, among the 51 genes with CNA loss lost in grade II/III but preserved in GBM primary cells, 46 were significantly correlated with patient survival (log-rank test, p<0.05). The HR of the alterations was <1 for one gene, *MIR1256*, and >1 for 45 genes, which were included in the gene with HR >1 with CNA loss lost in primary cells. Together, the results indicated that the gene CNAs lost in primary cells, regardless of gain or loss, were mainly good prognostic factors, which correlated to better survival, whereas gene CNAs preserved in primary cells predicted poor survival in glioma patients. We further tested the hypothesis with well-known molecular markers in glioma, including *IDH* mutation, 1p/19q codeletion, *TERT* promoter mutations, and *MGMT* promoter methylation. The detailed molecular information of the paired tumor and primary cells were listed in **Supplementary Table 6**. In grade II/III tissues, there were 6 out of 7 samples with *IDH* mutations, but none of the mutation were preserved in their primary cells (**Figure 6A**). Similar to *IDH* mutations, 1p/19q codeletion was present in 4 out of 7 grade II/III tissues, but all grade II/III primary cells were 1p/19q intact (**Figure 6B**). Moreover, *TERT* promoter mutations and methylated *MGMT* were lost in grade II/III primary cells, whereas GBM primary cells preserved most of the mutations and methylated *MGMT* (**Figures 6C, D**). Together, our results showed that most of the gene CNAs in grade II/III gliomas, which predicted better survival, were lost in their primary cells, whereas the GBM primary cells preserved the gene alterations in tumors, which predicted poor survival in glioma patients.

**Table 6:** The univariate Cox regression analysis of the association between overall survival of glioma patients and CNAs of targeted genes in the patient cohort from the Merged Cohort of LGG and GBM dataset.

Number of genes with CNA Gene status	Total	Total^#^(Genes in Merged Cohort of LGG and GBM)	OS difference^$$^ (P < 0.05)	Hazard Ratio^&^	
				<1 (Low risk)	>1 (High risk)
**Genes with CNA loss in patient specimens**					
Lost in primary cells	2424	2026	2015	1959	56^$^
Preserved in primary cells	802	660	540	9	531
**Genes with CNA gain in patient specimens**					
Lost in primary cells	7	4	4	0	4
Preserved in primary cells	847	648	647	0	647

^#^Total gene number with complete information of overall survival and copy number variation in the Merged Cohort of LGG and GBM dataset.

^$$^Overall survival difference between with or without specific copy number alterations. log-rank test.

^&^Hazard ratio. Patients with the alteration compared to patients without the alteration.

^$^Among the 56 genes with CNA loss and lost in glioma primary cells, there were 45 genes lost in the grade II/III primary cells but preserved in GBM primary cells.

## Discussion

The genetic alterations in tumors and their paired primary cells have been noticed and studied for decades. Previous researches mainly focused on the comparisons of the similarity and alteration between the parental tumor and the primary cell and identification of regions or genes with copy number altered on chromosomes (12, 13, 15). However, little is known about the correlation of these alterations to biological function or to disease. Here, we reported that the GBM primary cells preserved the parental tumor CNAs and these gene CNAs were correlated to poor survival in glioma patients. The CNAs in gliomas, such as chromosome 7 and 19 gains as well as 10 loss in primary GBMs, have been previously reported (5, 16, 17). In our study, GBM primary cells retained chromosome 7p gain and 10q loss from parental tumors, which is consistent with previous researches that GBM primary cells preserved the genetic alterations, amplifications on chromosome 7 and 20 as well as deletions on chromosome 9 and 10, in parental tumors (12). The chromosome 7p gain, including *EGFR* amplification, and 10q loss, including *PTEN* and *DMBT1* deletions, were often concurrent in GBMs (18, 19). The chromosome 10q loss has been identified to associate with shorter overall survival in high-grade glioma patients (19). The effect of the chromosome 7p amplification on glioma patient prognosis is not clear, but generally glioma patients with *EGFR* or chromosome 7p amplification have shorter survival (20–22). Moreover, we further identified chromosome 16p11 loss and amplifications of *ADAM6* and *LINC00226* as preserved genomic alterations in both GBM and grade II/III primary cells, whereas chromosome 3p21 loss only observed in grade II/III cells. *ADAM6* is often upregulated in cancers including glioma, and its expression was high in GBM and lower in ODG and correlated to poor outcome in lower-grade glioma (23). The expression of *LINC0026* was also correlated to patient survival, and up-regulation of *LINC00226* predicted poor prognosis and enhanced cell invasion, stemness, and proliferation in cancers (24–26). Although the correlation between cancer patient survival and chromosome losses in 16p11 and 3p21 regions are not clear, the CNAs in these regions are closely related to diseases. The 16p11 region possesses high allele frequencies of pathogenic CNAs often with deletions and is associated with cognitive and anatomical defects (27). The loss of chromosome 3p21, which harbors several tumor-suppressor genes, such as *BLU*, *RASSF1A*, and *SEMA3B*, are frequently observed in cancers including gliomas, and the 3p21.31 region is a female-specific risk locus in astrocytoma and GBM (28–31). Overall, our results and its consistency with known prognostic correlations support our hypothesis that the CNAs preserved in primary cells predicted poor prognosis in glioma patients and may identify novel molecular markers as potential prognostic factors in gliomas.

Current understanding of primary cells from grade II/III gliomas is limited as most of the primary cell researches have focused on GBMs. In our study, the grade II/III glioma primary cells lost most of the CNAs, which mainly consisted of chromosome deletions located on 1p and 19q regions. The chromosome 1p/19q codeletion, an ODG molecular marker, is positively correlated to better response to therapy as well as improved prognosis and overall survival in the WHO grade II/III patients (5, 32). Similar to the 1p/19q codeletion, *IDH* mutations are also important classification and prognostic factors in gliomas. The IDH1-R132H mutation is involved in the early gliomagenesis; however, after carcinogenesis complete, it is considered a radiosensitizing gene and a better prognostic factor that predicts favorable outcome in glioma patients (3, 33–35). Also, the mutation is associated with cellular and biological functions that impede cell growth, survival, and invasion in cultured glioma cells (36). In our study, 6 out of 7 grade II/III parental tumors possessed *IDH* mutations, but none of the mutations were preserved in their primary cells. The grade II/III primary cells may tend to lose CNAs or mutations that correlate to good prognosis in gliomas, as *IDH* mutations and 1p/19q codeletion were frequently found in grade II/III gliomas but absent in primary cells from grade II/III gliomas used in this study and other commonly used glioma cell lines. However, the chromosome deletion in the 10q22 region, which is correlated to poor survival as described previously, was lost in grade II/III primary cells when preserved in GBM primary cells. Similarly, *MGMT* promoter methylation and *TERT* promoter mutations, both well-known molecular markers correlated to drug-resistance in gliomas, were also found to be preserved in GBM primary cells but lost in grade II/III primary cells. Methylated *MGMT* promoter predicts a favorable response of GBM patients to alkylating chemotherapy and other studies suggested that patients with methylated *MGMT* promoter have better prognosis and survival (8, 9, 37). *TERT* promoter mutations, found in more than 70% of primary GBMs and ODGs, enhances the telomerase activity and tumor growth and is associated with a poor outcome in GBMs (38, 39). However, in grade II/III gliomas, *TERT* promoter mutation serves as a favorable prognostic factor similar to *IDH* mutations (38). Overall, the preservation status of molecular markers in primary cells were correlated with its prognostic relation to patient survival, and GBM primary cells seems to have the ability to allow more genetic alterations in their genome.

In this report, we isolated 11 primary cell lines from gliomas with different grades and histology, and analyzed genomic profiles of the primary cell lines and their parental tumors. As the incidence rate of glioma was relatively low among cancers and the fresh patient specimens suitable for primary culture were not easily accessible, we only successfully established 3 astrocytoma, 4 ODG, and 4 GBM primary cells with qualified CNV profiles. Sample size is a crucial factor towards the power to detect variants at different effect levels. A small sample size may limit detection to variants of large effects, whereas increasing the number of samples will not only enable detection of small effect variants, but also contribute to more detailed genetic analyses and convincing results for each glioma histology group. Here, we demonstrated that the culturing of primary cells may provide a potential method for selecting novel prognostic markers in gliomas. Factors predicting better prognosis in patients with brain cancer, such as *IDH* mutations and 1p/19q codeletion, were often lost in the grade II/III primary cells, whereas those that predicted poor outcome, such as chromosome 7p gain and10q loss, were preserved in the GBM primary cells. We report that GBM primary cells preserved most of the genomic alterations exhibited in its parental tumors, which were correlated to poor survival in glioma patients.

## Conclusion

We demonstrated the comparison of the genomic alterations between parental tumors and their primary cells identified several chromosome regions with CNA preserved, such as chromosome 7p gain, 10q loss, and 16p11 loss, or lost, such as 1p/19q codeletion in primary cells. The GBM primary cells preserved most of the alterations while grade II/III gliomas cells lost most of the alterations in parental tumors. The gene CNVs preserved in the primary cells were correlated to poor overall survival of glioma patients in the Merged Cohort of LGG and GBM dataset from cBioloPortal. In contrast, genomic alterations that were lost in grade II/III primary cells, such as 1p/19q codeletion and IDH mutations, were often associated to favorable outcome. Our results suggest that the preservation status of gene CNAs in primary cells were associated to their effects on patient survival, and GBM primary cells preserved the most gene CNAs that predicted poor survival in glioma patients.

## Data Availability Statement

The original contributions presented in the study are included in the article/**Supplementary Material**. Further inquiries can be directed to the corresponding author.

## Ethics Statement

The studies involving human participants were reviewed and approved by Chang Gung Medical Foundation Institutional Review Board. The patients/participants provided their written informed consent to participate in this study.

## Author Contributions

Y-JL and K-CW performed the surgical resection and sample collection. C-HL, L-YF, and C-HC participated in primary cell preparation, DNA extraction, and biomarker expression detection. C-HC and Y-YL performed the data collection, result analysis, and interpretation. C-HC and Y-JL participated in manuscript preparation. C-YH, K-CW, and IC planned and supervised the entire study. All authors read and approved the final version of the manuscript.

## Funding

This work was supported by the Chang Gung Memorial Hospital, Linkou Medical Center by CMRPG3H1141 to Y-JL; and the Ministry of Science and Technology, R.O.C. by grant number MOST 108-2314-B-182-019-MY2 and the National Health Research Institutes, Taiwan by grant number NHRI-EX110-10502NI to K-CW.

## Conflict of Interest

The authors declare that the research was conducted in the absence of any commercial or financial relationships that could be construed as a potential conflict of interest.

## Supplementary Material

Supplementary Table 1 STR profile of tumors and tumor derived primary cells.

Supplementary Table 2 The preserved and lost gene CNAs in primary cells from grade II/III gliomas.

Supplementary Table 3 The preserved and lost gene CNAs in primary cells from GBM tumors.

Supplementary Table 4 The molecular and cellular functions significantly correlated to the genes with CNA preserved or lost in primary cells.

Supplementary Table 5 The disease and biological functions significantly correlated to the genes with CNA preserved or lost in primary cells.

Supplementary Table 6 The status of IDH mutation, 1p/19q codeletion, TERT promoter mutation, and MGMT promoter methylation in paired tumors and primary cells.

Supplementary Figure 1 The prediction of the biological function status in grade II/III CNA-preserved and GBM CNA-preserved groups were shown and sized by activation z-score. Each area represented a disease related biological function was colored by z-score. The blue area indicated a decreasing function was expected and the orange indicated an increased function.

## Detection of IDH1/2 mutations

A 481 base pairs (bp) IDH1 fragment was amplified by the primer set: 5’-TGCCACCAACGACCAAGTCA-3’ (forward) and 5’-GTGTTGAGATGGACGCCTAT TTG-3’ (reverse) and a 290 bp IDH2 fragment was amplified by primers: 5’-GGGGTTCAAATTCTGGTTGA-3’ (forward) and 5’-CTAGGCGAGGAGCTCCAGT-3’ (reverse). The PCR reactions were performed in a total volume of 20 μl solution containing ~200 ng DNA, 100 nM primers, and 1X GoTaq Green Master Mix (M712, Promega). The PCR cycling conditions were set at 94°C for 1 minute, 55°C 30 seconds, and 72°C for 1 minute for 40 cycles. A second PCR was performed using 1 μl product from first PCR with 100 nM primer sets: 5’-CAGGAAACAGCTATGACCATGATTACGTGCCACCAACGACCAAGTCA-3’ (forward) and 5’-CGTTGTAAAACGACGGCCAGTGAATTGTGTGTTGAGATGGACGCCTATTTG-3’ (reverse) for IDH1 or 5’-CAGGAAACAGCTATGACCATGATTACGGGGGTTCAAATTCTGGTTGA-3’ (forward) and 5’-CGTTGTAAAACGACGGCCAGTGAATTGCTAGGCGAGGAGCTCCAGT-3’ (reverse) for IDH2, and 1X CleanAmp GC-Rich Master Mix (L-5102, TriLink) in a total volume of 20 μl. The PCR cycling condition was same with described above. The final product was sequenced using primer 5’-CAGGAAACAGCTATGACCATGATTACG-3’ or 5’-CGTTGTAAAACGACGGCCAGTGAATTG-3’.

## Detection of TERT promoter mutations

The PCR reactions were performed in a total volume of 20 μl solution containing ~200 ng DNA, 100 nM primers, and 1X GoTaq Green Master Mix (M712, Promega). The PCR cycling conditions were set at 94°C for 1 minute, 63°C 30 seconds, and 72°C for 1 minute for 35 cycles. A 244 bp DNA fragment was amplified by the primer set: 5’- GCACAGACGCCCAGGACCGCGCT -3’ (forward) and 5’- TTCCCACGTGCGCAGCAGGACGCA -3’ (reverse). A second PCR was performed using 1 μl product from first PCR with 100 nM primer sets: 5’-CAGGAAACAGCTATGACCATGATTACG GCACAGACGCCCAGGACCGCGCT -3’ (forward) and 5’-CGTTGTAAAACGACGGCCAGTGAATTGTTCCCACGTGCGCAGCAGGACGCA -3’ (reverse), and 1X CleanAmp GC-Rich Master Mix (L-5102, TriLink) in a total volume of 20 μl. The PCR cycling condition was same with described above. The final product was sequenced using primer 5’-CAGGAAACAGCTATGACCATGATTACG-3’ or 5’-CGTTGTAAAACGACGGCCAGTGAATTG-3’.
