# Supplementary material for: Glioblastoma Primary Cells Retain the Most Copy Number Alterations That Predict Poor Survival in Glioma Patients
Source: Front Oncol. 2021 Apr 26;11:621432. doi: 10.3389/fonc.2021.621432 (PMC8108987; doi:10.3389/fonc.2021.621432)
Supplement: Supplementary Table 1 — STR profile of tumors and tumor derived primary cells. [file DataSheet_1.pdf]

Supplementary Table 1. STR profile of tumors and tumor-derived primary cells

STR markers	D8S1179	D21S11	D7S820	CSF1PO	D3S1358	TH01	D13S317	D16S539	D2S1338	D19S433	vWA	TPOX	D18S51	D5S818	FGA	Amelo.
W802T	12/15	29/30	11/11	12/12	15/17	7/9	10/13	10/12	19/20	13/14	16/18	8/11	14/21	12/12	23/24	XY
W802C	12/15	29/30	11/11	12/12	15/17	7/9	10/13	10/12	19/20	13/14	16/18	8/11	14/21	12/12	23/23	XY
W909T	10/11	28/30	8/10	12/13	15/16	7/10	8/9	12/13	24/24	13/15.2	16/16	8/12	14/15	10/10	25/26	XY
W909C	10/11	28/30	8/10	12/13	15/16	7/10	8/9	12/13	24/24	13/13	16/16	8/8	15/15	10/10	25/26	XY
W919T	13/14	29/33.2	12/12	12/12	16/17	9/9	11/12	9/9	19/22	14/16.2	14/16	8/11	16/19	7/12	22/23	XX
W919C	13/14	29/33.2	12/12	12/12	16/17	9/9	11/12	9/9	19/22	14/16.2	14/16	8/11	16/19	7/12	22/23	XX
W928T	9/15	28/29	11/12	10/12	16/16	9/9	7/10	12/13	23/24	12.2/15	17/17	8/11	14/16	9/12	19/21	XX
W928C	9/15	28/29	11/12	10/12	16/16	9/9	7/10	12/13	23/24	12.2/15	17/17	8/11	14/16	9/12	19/21	XX
W933T	13/15	29/30	8/9	12/12	15/16	9/10	8/8	10/11	21/24	14/15	15/16	8/8	13/18	11/11	19/22	XY
W933C	13/15	29/30	8/9	12/12	15/16	9/10	8/8	10/11	21/24	14/15	15/16	8/8	13/18	11/11	19/22	XY
W935T	11/13	30/32.2	8/12	10/11	15/16	9/9	8/9	9/9	23/24	13/14.2	14/19	11/11	16/16	10/12	22/24	XX
W935C	11/13	30/32.2	8/12	10/11	15/16	9/9	8/9	9/9	23/24	13/14.2	14/19	11/11	16/16	10/12	24/24	XX
W937T	11/15	30.2/31	10/12	11/11	15/17	9/9	9/9	11/13	20/23	13/13	14/19	11/12	14/20	10/13	19/19	XX
W937C	11/15	30.2/31	10/12	11/11	15/17	9/9	9/9	11/13	20/23	13/13	14/19	11/12	14/20	10/13	19/19	XX
W946T	10/15	28/32.2	11/12	10/10	15/15	7/9	11/12	9/11	20/23	13.2/14	14/16	8/11	13/15	10/10	22/26	XY
W946C	10/15	28/32.2	11/12	10/10	15/15	7/9	11/12	9/11	20/23	13.2/14	14/16	8/11	13/15	10/10	22/26	XY
W950T	12/13	30/31	11/11	12/12	15/16	9/9	9/13	9/12	16/24	13.2/13.2	14/14	8/11	12/13	9/12	18/24	XX
W950C	12/13	30/31	11/11	12/12	15/16	9/9	9/13	9/12	16/24	13.2/13.2	14/14	8/11	12/13	9/12	18/24	XX
W952T	11/13	28.2/31	9/11	10/11	15/17	6/8	10/10	11/12	23/24	14/15.2	16/17	8/8	15/17	10/11	22/24	XY
W952C	11/13	28.2/31	9/11	10/11	15/17	6/8	10/12	11/12	23/24	14/15.2	16/17	8/8	15/17	10/11	22/24	XY
W958T	11/12	32/32.2	8/12	12/13	15/15	9/10	8/10	9/11	18/19	14/16.2	17/18	8/12	14/15	10/12	18/21	XY
W958C	11/12	32/32.2	8/12	12/12	15/15	9/10	8/8	9/11	18/19	14/16.2	17/18	8/12	14/15	10/10	18/21	XY
