# Supplementary material for: Glioblastoma Primary Cells Retain the Most Copy Number Alterations That Predict Poor Survival in Glioma Patients
Source: Front Oncol. 2021 Apr 26;11:621432. doi: 10.3389/fonc.2021.621432 (PMC8108987; doi:10.3389/fonc.2021.621432)
Supplement: Supplementary Table 6 — The status of IDH mutation, 1p/19q codeletion, TERT promoter mutation, and MGMT promoter methylation in paired tumors and primary cells. [file Presentation_1.pptx]

## Slide 1
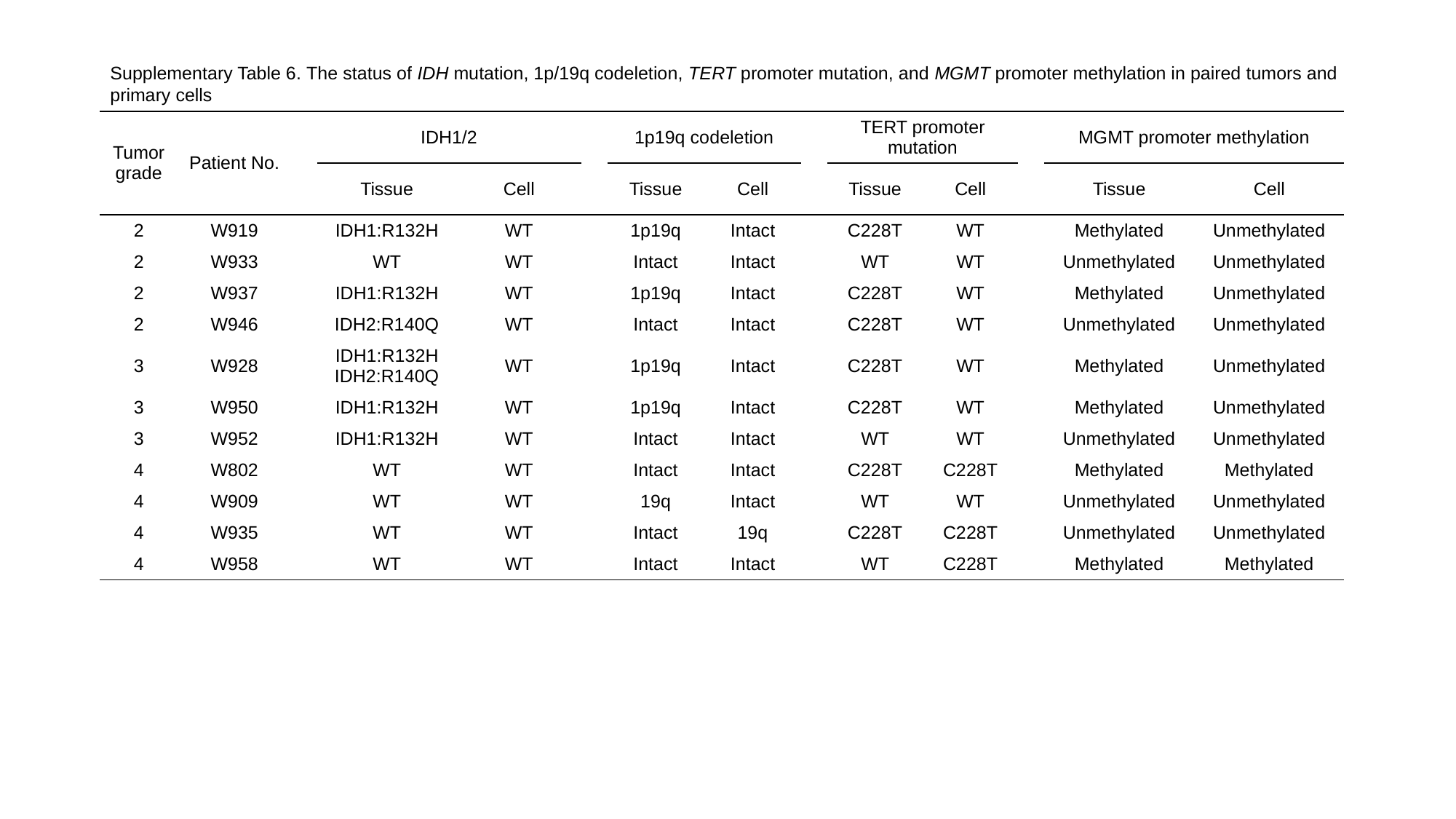

Supplementary Table 6. The status of IDH mutation, 1p/19q codeletion, TERT promoter mutation, and MGMT promoter methylation in paired tumors and primary cells
| Tumor grade | Patient No. | | IDH1/2 | | | 1p19q codeletion | | | TERT promoter mutation | | | MGMT promoter methylation | |
| --- | --- | --- | --- | --- | --- | --- | --- | --- | --- | --- | --- | --- | --- |
| | | | Tissue | Cell | | Tissue | Cell | | Tissue | Cell | | Tissue | Cell |
| 2 | W919 | | IDH1:R132H | WT | | 1p19q | Intact | | C228T | WT | | Methylated | Unmethylated |
| 2 | W933 | | WT | WT | | Intact | Intact | | WT | WT | | Unmethylated | Unmethylated |
| 2 | W937 | | IDH1:R132H | WT | | 1p19q | Intact | | C228T | WT | | Methylated | Unmethylated |
| 2 | W946 | | IDH2:R140Q | WT | | Intact | Intact | | C228T | WT | | Unmethylated | Unmethylated |
| 3 | W928 | | IDH1:R132H IDH2:R140Q | WT | | 1p19q | Intact | | C228T | WT | | Methylated | Unmethylated |
| 3 | W950 | | IDH1:R132H | WT | | 1p19q | Intact | | C228T | WT | | Methylated | Unmethylated |
| 3 | W952 | | IDH1:R132H | WT | | Intact | Intact | | WT | WT | | Unmethylated | Unmethylated |
| 4 | W802 | | WT | WT | | Intact | Intact | | C228T | C228T | | Methylated | Methylated |
| 4 | W909 | | WT | WT | | 19q | Intact | | WT | WT | | Unmethylated | Unmethylated |
| 4 | W935 | | WT | WT | | Intact | 19q | | C228T | C228T | | Unmethylated | Unmethylated |
| 4 | W958 | | WT | WT | | Intact | Intact | | WT | C228T | | Methylated | Methylated |
