# Supplementary material for: Glioblastoma Primary Cells Retain the Most Copy Number Alterations That Predict Poor Survival in Glioma Patients
Source: Front Oncol. 2021 Apr 26;11:621432. doi: 10.3389/fonc.2021.621432 (PMC8108987; doi:10.3389/fonc.2021.621432)
Supplement: Supplementary Figure 1 — The prediction of the biological function status in grade II/III CNA-preserved and GBM CNA-preserved groups were shown and sized by activation z-score. Each area represented a disease related biological function was colored by z-score. The blue area indicated a decreasing function was expected and the orange indicated an increased function. [file DataSheet_2.pdf]

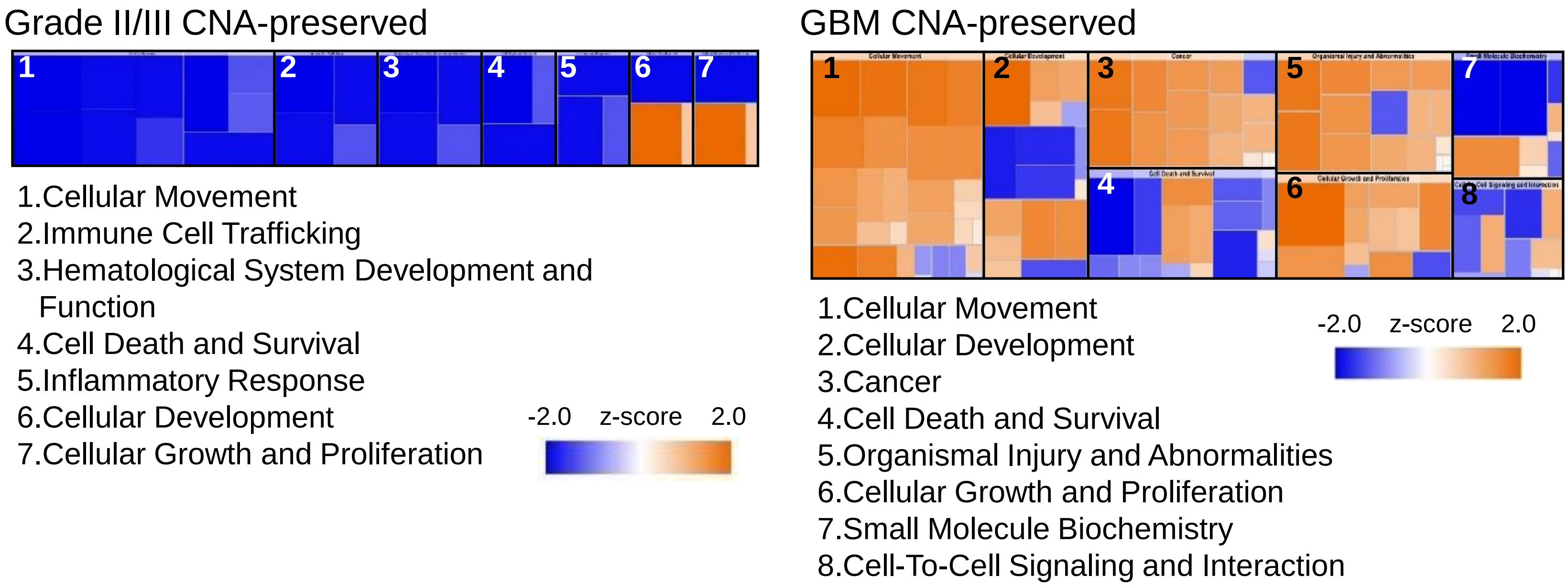
Grade II/III CNA-preserved
1
2
3
4
5
6
7
1.Cellular Movement
2.Immune Cell Trafficking
3.Hematological System Development and Function
4.Cell Death and Survival
5.Inflammatory Response
6.Cellular Development
7.Cellular Growth and Proliferation
-2.0 z-score 2.0
GBM CNA-preserved
1
2
3
4
5
6
7
8
1.Cellular Movement
2.Cellular Development
3.Cancer
4.Cell Death and Survival
5.Organismal Injury and Abnormalities
6.Cellular Growth and Proliferation
7.Small Molecule Biochemistry
8.Cell-To-Cell Signaling and Interaction
-2.0 z-score 2.0
